# Age-Related Regional Network Covariance of Magnetic Resonance Imaging Gray Matter in the Rat

**DOI:** 10.3389/fnagi.2020.00267

**Published:** 2020-08-26

**Authors:** Gene E. Alexander, Lan Lin, Eriko S. Yoshimaru, Pradyumna K. Bharadwaj, Kaitlin L. Bergfield, Lan T. Hoang, Monica K. Chawla, Kewei Chen, James R. Moeller, Carol A. Barnes, Theodore P. Trouard

**Affiliations:** ^1^Department of Psychology, University of Arizona, Tucson, AZ, United States; ^2^Department of Psychiatry, University of Arizona, Tucson, AZ, United States; ^3^Evelyn F. McKnight Brain Institute, University of Arizona, Tucson, AZ, United States; ^4^Neuroscience Graduate Interdisciplinary Program, University of Arizona, Tucson, AZ, United States; ^5^Physiological Sciences Graduate Interdisciplinary Program, University of Arizona, Tucson, AZ, United States; ^6^Arizona Alzheimer’s Consortium, Phoenix, AZ, United States; ^7^Department of Biomedical Engineering, University of Arizona, Tucson, AZ, United States; ^8^Division of Neural Systems, Memory and Aging, University of Arizona, Tucson, AZ, United States; ^9^Banner Samaritan PET Center and Banner Alzheimer’s Institute, Banner Good Samaritan Medical Center, Phoenix, AZ, United States; ^10^Department of Psychiatry, Vagelos College of Physicians and Surgeons, Columbia University Irving Medical Center, Columbia University, New York, NY, United States; ^11^Department of Neurology, University of Arizona, Tucson, AZ, United States; ^12^Department of Neuroscience, University of Arizona, Tucson, AZ, United States

**Keywords:** aging, behavior, prefrontal cortex, perirhinal cortex, structural covariance, scaled subprofile model

## Abstract

Healthy human aging has been associated with brain atrophy in prefrontal and selective temporal regions, but reductions in other brain areas have been observed. We previously found regional covariance patterns of gray matter with magnetic resonance imaging (MRI) in healthy humans and rhesus macaques, using multivariate network Scaled Subprofile Model (SSM) analysis and voxel-based morphometry (VBM), supporting aging effects including in prefrontal and temporal cortices. This approach has yet to be applied to neuroimaging in rodent models of aging. We investigated 7.0T MRI gray matter covariance in 10 young and 10 aged adult male Fischer 344 rats to identify, using SSM VBM, the age-related regional network gray matter covariance pattern in the rodent. SSM VBM identified a regional pattern that distinguished young from aged rats, characterized by reductions in prefrontal, temporal association/perirhinal, and cerebellar areas with relative increases in somatosensory, thalamic, midbrain, and hippocampal regions. Greater expression of the age-related MRI gray matter pattern was associated with poorer spatial learning in the age groups combined. Aging in the rat is characterized by a regional network pattern of gray matter reductions corresponding to aging effects previously observed in humans and non-human primates. SSM MRI network analyses can advance translational aging neuroscience research, extending from human to small animal models, with potential for evaluating mechanisms and interventions for cognitive aging.

## Introduction

Healthy aging in humans has been associated with brain atrophy on magnetic resonance imaging (MRI) scans often involving prefrontal and selective temporal brain regions. Atrophy in other brain areas, however, has been widely observed, including in parietal cortex, subcortical white matter, and cerebellum (Raz et al., 1998; Good et al., 2001; Tisserand et al., 2002; Alexander et al., 2006; Raz and Rodrique, 2006). Studies of human cognitive aging have shown differences between young and older adults in cognitive abilities often thought to be mediated by structures impacted by brain aging, with aspects of memory, executive function, and processing speed preferentially affected (Tisserand and Jolles, 2003; Park and Reuter-Lorenz, 2009; Alexander et al., 2012a). Such human studies, however, often do not exclude common health conditions of aging, like hypertension, that can contribute to both structural brain changes and the associated cognitive decline (Yoshita et al., 2005). Also, these studies typically cannot exclude the potential for developing neurodegenerative disease pathology, e.g., Alzheimer’s disease, in which the clinical symptoms have not yet appeared. Studying the effects of aging on MRI brain structure in non-human animal models provides the opportunity to investigate how the aging process impacts the brain and related cognitive functions without the potential complications of concurrent human health conditions, associated medications, and the possibility of incipient neurodegenerative disease. The importance of regional brain networks in supporting cognition has been well established (Goldman-Rakic, 1988). Evaluating alterations of age-related brain networks may be particularly relevant for understanding how aging affects cognitive functions over the lifespan (Burke and Barnes, 2006).

We previously found MRI gray matter network patterns in healthy humans (Alexander et al., 2006; Bergfield et al., 2010) and in a non-human primate, rhesus macaque model of healthy aging (Alexander et al., 2008) using multivariate regional covariance analysis, the Scaled Subprofile Model (SSM; Moeller et al., 1987; Alexander and Moeller, 1994), with voxel-based morphometry (VBM; Ashburner and Friston, 2000). These research studies indicated reductions in the prefrontal cortex and selective temporal brain regions as common regional features of aging shared across humans and a non-human primate species. Multivariate covariance methods, like SSM, can identify how brain regions interact in the context of aging or disease related to alterations in functional and anatomical associations (Alexander and Moeller, 1994; Alexander-Bloch et al., 2013). SSM analysis has been utilized in multiple studies in neuroimaging research of brain function and structure (Alexander and Moeller, 1994; Eidelberg et al., 1995; Moeller and Eidelberg, 1997; Alexander et al., 1999, 2008, 2012b; Habeck et al., 2003; Smith et al., 2006; Brickman et al., 2007), but has yet to be applied to neuroimaging scans in studies of brain aging with rodents. The ability to link findings observed in human and non-human primates with that of rodent models of aging, by using common neuroimaging methods across species, provides a unique opportunity to advance translational cognitive aging research. The application of such cross-cutting neuroimaging techniques that can scale from human to small animal models, has the potential to advance our understanding of the mechanisms underlying human brain aging and how putative interventions can lead to enhanced cognitive performance.

In the current study, we investigated the effect of aging on the regional covariance of MRI gray matter in 10 young and 10 aged male Fischer 344 rats using SSM with VBM to identify the regional age-related network pattern of gray matter in this rodent model of healthy aging. We hypothesized: (1) that brain areas affected in our previous studies of healthy aging in humans and rhesus macaques, including prefrontal and temporal regions, would also show reductions in aged rats as part of an age-related regional pattern of gray matter volume; and (2) that greater expression of the age-related pattern among the rats would be associated with diminished learning performance on the spatial Morris swim task (Morris, 1984), an ability affected in aged rodents with linkages to human cognitive aging (Alexander et al., 2012a).

## Materials and Methods

### Subjects and Behavioral Testing Procedures

This study included 10 young (mean ± SD age = 10.8 ± 0.9 months; range = 9.6–11.7 months old) and 10 aged (mean ± SD age = 25.2 ± 0.7 months; range = 24.1–26.2 months old) Fischer 344 male rats. All behavioral and scanning procedures were performed following rodent guidelines from the National Institutes of Health and were conducted under the University of Arizona Institutional Animal Care and Use Committee approved protocols. All rats participated in study procedures in pairs that included one young and one aged rat. In this case, every single young-old pair arrived in the colony in the same animal batch and underwent the same behavioral procedures during the same days. The rats were maintained on a 12-h light/dark cycle and were housed individually. Before testing, the rats were handled over several days, at least 5–10 min per day, by experimenters. Testing of the rats was performed with spatial and visually cued versions of the Morris swim task (Morris, 1984). Briefly, during the spatial version of the task, the water was made opaque to hide the escape platform beneath the surface. The rats were tested with six trials per day over four consecutive days.

After the spatial trials were administered, the rats were screened for their visual ability over 2 days with six trials per day of the cued visual task. In this case, the platform was visible above the water’s surface. The platform’s position was moved between each trial (Barnes et al., 1996). Performances of the rats on the swim tasks were analyzed using standard software (WMAZE, M. Williams, or ANY-maze). Since varying release locations and differences in swim velocity lead to variability in the time to reach the escape platform, a corrected integrated path length (CIPL) was computed for comparability of the animals’ performance for the differing locations of release (Gallagher et al., 2015). The CIPL values measure the cumulative distance from the escape platform over time, corrected for a rat’s swim velocity. CIPL is equivalent to the previously described cumulative search error (Gallagher et al., 2015). Also, a CIPL learning index was calculated for each rat by subtracting Day 4 average performance from Day 1 average performance and dividing by the sum of the 2 days.

### Image Acquisition

Volumetric T2-weighted MRI scans were acquired on a 7T horizontal bore Bruker Biospec Instrument (Bruker BioSpin) equipped with self-shielded gradients capable of 600 mT/m with 125 ms rise times. A 72 mm ID birdcage coil was used for excitation and a four-element phased-array surface coil was used for reception. All animals were scanned in the prone position using an animal bed restraint system including ear bars and a bite bar for fixation of the head. The rats received anesthesia with 1.5% isoflurane gas, their breathing rates were monitored, and body temperature was monitored and maintained at 37 ± 1°C with a heated circulating water system. Anatomical brain images were acquired with 150-μm isotropic voxel resolution using a three dimensional Fast Spin-Echo (3DFSE) sequence and the following scan parameters: relaxation time (TR) = 1,500 ms, echo time (TE) = 40 ms effective, flip angle = 90°, matrix = 256 × 192 × 128, field of view (FOV) = 3.84 × 2.88 × 1.92 cm.

### Image Processing

Processing of the MRI scans was performed with Statistical Parametric Mapping (SPM8; Wellcome Trust Centre for Neuroimaging, London, UK) using voxel-based morphometry (Ashburner and Friston, 2000; Good et al., 2001) with DARTEL for diffeomorphic anatomical inter-subject registration and to generate segmented gray matter maps (Ashburner, 2007; Ashburner and Friston, 2009) for subsequent SSM analysis. First, scans were processed using MRIcro software (Rorden and Brett, 2000) to manually segment the whole brain from surrounding non-brain tissue. A single rater (LL) performed this “skull stripping” procedure by manually tracing the brain area for each MRI section while being viewed in three dimensions and then combining all traced scan slices to create a whole-brain mask for segmentation of the non-brain tissue. High inter-rater and intra-rater reliabilities (0.98 and 0.96, respectively) for this procedure have been previously reported (Alexander et al., 2008) and intra-rater reliability for a subset of 12 rat-MRI scans in this study was 0.99. N3 software (Sled et al., 1998) corrected for field inhomogeneity on the masked rat brain images and VBM tissue prior probability maps were constructed through an iterative procedure (Alexander et al., 2008). Briefly, a representative rat brain from the sample was manually segmented for gray, white, and cerebrospinal fluid tissue and histogram shape-based image thresholding was performed and applied to the selected rat brain MRI to determine optimal values to segment the three tissue classes (Otsu, 1979). VBM segmentation was then performed using these initial priors to segment each of the 20 rat MRI scans. An average of the segmented brain maps for all subjects was obtained to produce a new set of initial priors and this VBM segmentation and prior generation step was repeated iteratively until the difference between the current and subsequent priors reached convergence at <0.1. SPM8 was subsequently used to produce gray and white matter maps using the generated priors as the seed point for use in the DARTEL routine, which simultaneously registers the scans, generating templates in the sample’s average MRI space, that is iteratively performed with increasing accuracy. The iterative procedure includes non-linear spatial normalizations that map the individual brain scan to a set of increasingly refined templates created over the sample of all subjects. Each map of gray matter was processed to preserve volume information in the context of spatial deformation and was smoothed using a 600-μm Gaussian kernel for statistical analysis. Visual inspection was performed during all image processing procedures to evaluate image quality at each step and processing was performed blind to group membership. A total intracranial volume estimate (eTIV) was computed by re-combining the segments of gray matter, white matter, and cerebrospinal fluid for each subject, in their native MRI brain scan space.

### Statistical Analysis of Imaging and Behavioral Performance

Regional network analysis with SSM was performed using MATLAB (MathWorks) on the smoothed gray matter maps produced by the MRI VBM processing. The underlying assumptions of the SSM have been described (Moeller et al., 1987; Alexander and Moeller, 1994). Briefly, after natural log transformation, the means across subjects and regions were subtracted at each image voxel of the smoothed maps of gray matter volume. A principal component analysis (PCA) was then performed to produce regional covariance patterns and their corresponding subject scores that reflect the degree to which each rat expressed the identified component network patterns. SSM was performed on the gray matter volume maps to identify network covariance patterns whose subject scores differed between the groups of young and aged rats. As such, each rat’s network score reflects individual differences in the degree to which they express the shared age-related SSM gray matter pattern that was determined from the young and old groups combined. We used multiple regression analysis to identify the best set of SSM network component patterns that distinguished the young from old age groups. We applied the Akaike Information Criterion, adjusted for application with small samples (AICc; Burnham and Anderson, 2002), as a method for model selection that optimizes the trade-off between bias and variance explained in identifying the best set of SSM components in a regression model (Habeck et al., 2005; Alexander et al., 2012b). Regression analysis was additionally performed to test the effect of differences in total intracranial size, using eTIV as a covariate, on the age group-related network pattern of gray matter. Network subject scores for the age-related SSM pattern were additionally tested in relation to performance on the spatial Morris swim task using the CIPL learning index.

We used bootstrap resampling (Efron and Tibshirani, 1994) performed with 500 iterations for the SSM analysis as described previously (Alexander et al., 2008, 2012b; Bergfield et al., 2010), providing reliability estimates at each voxel for the age-related SSM regional gray matter pattern weights. The calculated bootstrap distributions reflect confidence intervals for the network component voxel weights of the SSM pattern whose subject scores distinguished the young from aged groups. The local minima and maxima values for the bootstrapped SSM network pattern were selected with conservative thresholds of *z*-scores ≥ +3.5 or ≤ −3.5 and a spatial extent threshold of 50 voxels to show the major contributing brain regions in the age-related, network pattern. Two raters (PB and GA) reviewed and discussed to consensus the localization of the key regions from the SSM network pattern identified using the rat brain atlas by Paxinos and Watson (2007). This procedure included a systematic review of each slice in the averaged MRI brain template overlaid by the bootstrapped and thresholded SSM age-related pattern to localize the major regions characterizing the network covariance pattern, which was consistent with the approach previously applied for the MRI SSM analysis of aging in a non-human primate model (Alexander et al., 2008).

Age group differences in the visual Morris swim task, for each day of testing, were evaluated with non-parametric Mann–Whitney *U* tests. Analysis of the rats’ behavioral performance on the spatial Morris swim task was tested with a mixed-design analysis of covariance (ANCOVA) model. For this mixed model ANCOVA, performance on the visual swim task averaged across 2 days was included as an initial covariate, age group was the between-subjects factor, and days of testing was the within-subjects factor. The normality of the variables and samples included in the regression and mixed model ANCOVA analyses were confirmed with Shapiro–Wilk (S–W) tests (S–W statistics ≥ 0.85, *p*-values > 0.05).

## Results

### Behavioral Characterization of the Sample

**Figure 1:**
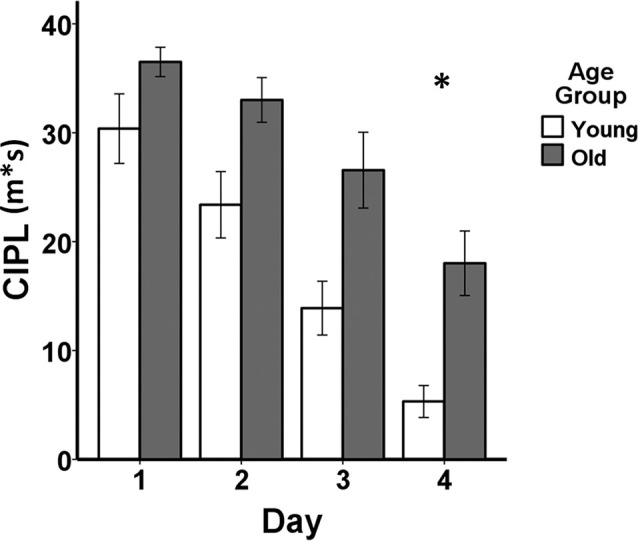
Behavioral performance comparing young adult (*n* = 10) and aged (*n* = 10) Fischer 344 male rats on the spatial Morris swim task. The bar graph shows means and standard error of the means (SEM) in the young (white bars) and old (gray bars) groups for the spatial swim task for each of the four consecutive days of testing indicated on the x-axis. Performance on the corrected integrated path length (CIPL) measured in meters*seconds (m*s) is shown on the y-axis. *After adjusting for visual swim task performance averaged across 2 days of testing, a mixed-design analysis of covariance (ANCOVA) tested for age group, days of testing, and age group × days of testing interaction effects for the spatial swim task. The aged rats showed poorer spatial learning compared to the young adult rats (*p* = 0.036) and both groups showed increasingly shorter CIPL distances traveled over the 4 days of testing (*p* = 1.30E-6), but there was no group by days of testing interaction (*p* = 0.48).

Behavioral performance on the spatial Morris swim task in the young and aged rats is shown in Figure 1. Non-parametric Mann–Whitney (M–W) *U* tests revealed that the aged rats (Mean rank = 14.20) demonstrated poorer performance on day 1 of the visual swim task (M–W *U* = 13.00, *p* ≤ 0.005) compared to the young rats (Mean rank = 6.80), but did not differ on day 2 (young rats Mean rank = 8.50, old rats Mean rank = 12.50, M–W *U* = 30.00, *p* = 0.13). After entering the average of the 2 days of visual task performance as a covariate in the mixed ANCOVA, the difference in learning on the spatial Morris swim task was significant between the young and aged rats (*F*_(1,17)_ = 5.18, *p* = 0.036). Additionally, the effect of days of testing was significant (*F*_(3,51)_ = 13.52, *p* = 1.30E-6) and there was no group by days of testing interaction (*F*_(3,51)_ = 0.84, *p* = 0.48).

### Effect of Age Group on MRI Gray Matter

**Figure 2:**
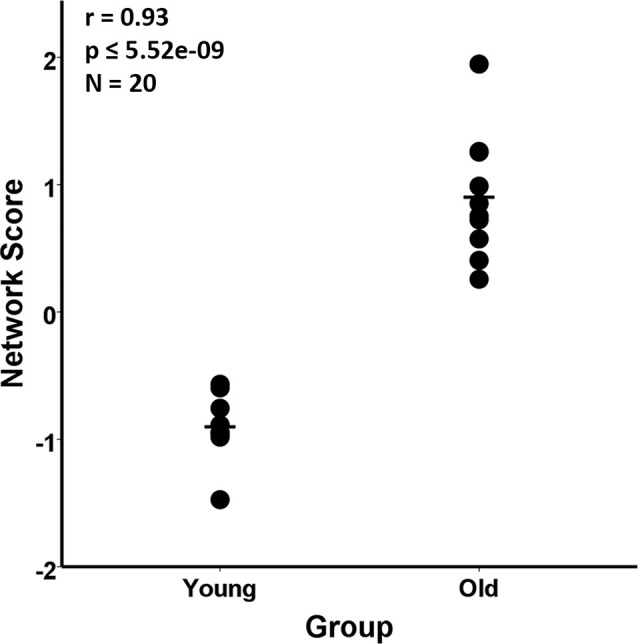
Scaled Subprofile Model (SSM) subject scores from the network analysis of magnetic resonance imaging (MRI) voxel-based morphometry (VBM) in young adults (*n* = 10) and aged (*n* = 10) Fischer 344 male rats. The scatterplot shows that the old group had a higher expression of the MRI age-related network pattern than the young group. The group difference between the young and aged rats in the SSM analysis of the MRI VBM gray matter maps was tested with a multiple regression model using the Akaike Information Criterion (AICc), which selected the first SSM component that accounted for 85.5% of the variance in distinguishing the young from aged groups (*r* = 0.93, *p* ≤ 5.52E-9).

The group difference between the young and aged rats in the MRI VBM SSM analysis of the gray matter maps was investigated with multiple regression using the AICc model selection method to identify the best set of pattern components differing between the groups. The model selected only the first SSM component, accounting for 85.5% of the variance in distinguishing the young from aged groups (*F*_(1,18)_ = 106.43, *p* = 5.52E-9; Figure 2). With eTIV added as an initial covariate to the regression model, the age group effect remained significant (*F_change_*_(1,17)_ = 106.03, *p* = 1.01E-8) and eTIV was not a significant predictor (*F*_(1,18)_ = 0.16, *p* = 0.69).

**Figure 3:**
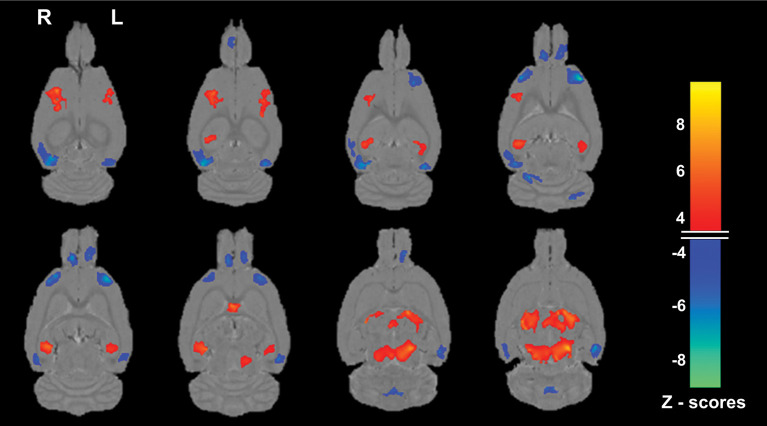
Magnetic resonance imaging (MRI) gray matter pattern reflecting the first Scaled Subprofile Model (SSM) component whose subject scores predicted age group in the Fischer 344 male rats. Voxels with SSM pattern weights are superimposed on horizontal slices from the statistical parametric mapping (SPM8) average MRI scans. The blue end of the color scale indicates brain regions showing lower gray matter volume with older age, whereas the orange end of the scale shows areas of relatively increased gray matter with increasing age. A rat with a high positive score for this age-related pattern has relatively greater reductions in the blue areas and relatively greater covarying increases in the orange areas. Only voxels with *z* scores ≤ −3.5 or ≥ + 3.5 and a 50 voxel extent after bootstrap re-sampling with 500 iterations to provide robust regional pattern weights are shown. Notable regions of reduction (blue) with older age are observed (from top-bottom rows) mainly in the vicinities of the bilateral temporal association/perirhinal, prefrontal/insula, olfactory bulb, and cerebellum; whereas relative increases (orange) are seen in the vicinities of the bilateral somatosensory, selected hippocampal, septal, thalamus, and midbrain regions. That all the aged rats had higher network age pattern scores than the young adult rats and in the positive range is consistent with a higher expression of the hypothesized age-related regional pattern in that group, including greater reductions in the blue areas than in the young.

The age-related SSM MRI VBM pattern with bootstrap resampling was characterized by relative gray matter volume reductions with voxel weights having maximum negative *z* scores mainly in vicinities of bilateral regions of the prefrontal/insula (−7.86 ≤ *z* ≤ −5.55), temporal association/perirhinal (−7.40 ≤ *z* ≤ −4.66), and olfactory bulb (−6.69 ≤ *z* ≤ −4.69) areas and the cerebellum (−7.62 ≤ *z* ≤ −5.81). Areas of relative increase with voxels having maximum positive *z* scores were observed in the vicinities of bilateral somatosensory (4.17 ≤ *z* ≤ 7.01), thalamic (4.23 ≤ *z* ≤ 8.22), midbrain (3.79 ≤ *z* ≤ 8.25), septal nuclei (*z* = 6.91) and selective hippocampal (3.96 ≤ *z* ≤ 6.55) regions (Figure 3).

### Association of Age-Related MRI Network of Gray Matter Volume With Behavior

**Figure 4:**
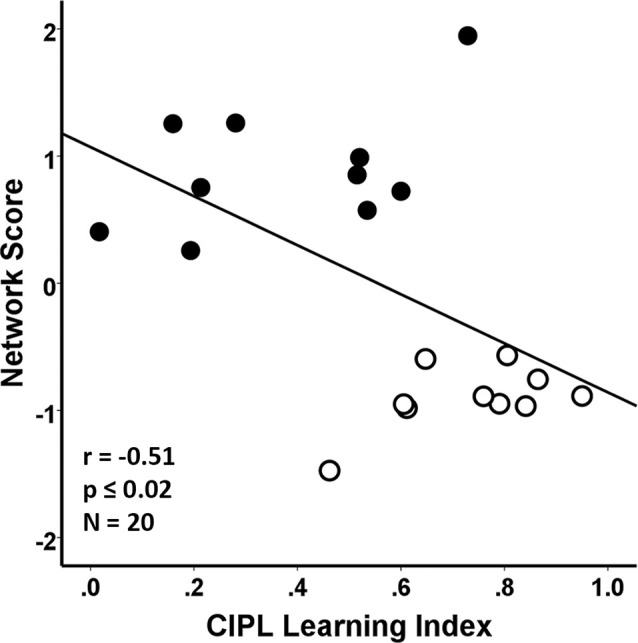
Regression analyses showing the association between behavioral performance on a corrected integrated path length (CIPL) learning index score of the spatial Morris swim task with the subject scores from the age-related network Scaled Subprofile Model (SSM) pattern. The learning index is derived from subtracting average CIPL performance on Day 4 from Day 1 and dividing by the sum of the 2 days. The aged rats are shown by filled circles and the young rats have open circles in the scatterplot. A higher expression of the network age pattern is associated with poorer task performance for both the young and old groups combined, accounting for 26% of the variance (*r* = −0.51, *p* ≤ 0.02), demonstrating that the age-related variance in the MRI network pattern is related to learning performance across the age-groups.

Performance on the CIPL learning index of the spatial Morris swim task was associated with the SSM gray matter network scores in the young and aged groups combined. In this case, poorer learning performance on the CIPL index was associated with a greater expression of the age-related SSM MRI VBM pattern and accounted for 26% of the variance (*F*_(1,18)_ = 6.29, *r* = −0.51, *p* = 0.02; Figure 4).

## Discussion

Aging in the rat was characterized by a regionally distributed MRI network pattern of gray matter reductions that includes brain regions in the prefrontal/insula and temporal association/perirhinal cortices, as well as the cerebellum and olfactory bulb. We used MRI at 7.0T on a voxel basis to evaluate the regional differences in gray matter volume between young adult and aged rats using a multivariate model of regional covariance, the SSM (Moeller et al., 1987; Alexander and Moeller, 1994), a method that has been applied in structural MRI studies of aging in humans (Alexander et al., 2006; Brickman et al., 2007, 2008; Bergfield et al., 2010) and also in rhesus macaques (Alexander et al., 2008). This study represents the first demonstration of a regional network covariance pattern of gray matter volume in a rodent model of aging, using SSM network analysis with high-resolution structural MRI. In this case, the SSM network regional pattern was computed for both the young and old rats combined, so the young and old animals expressed the same MRI regional gray matter pattern, but to different degrees, as reflected by the differences in the network scores between the groups. The findings suggest that aged rats experience reductions in gray matter in regions of prefrontal and selective temporal cortices and are consistent with previous work using the same multivariate analysis methods in humans and non-human primates, supporting common regional features of brain aging across species. The findings are also generally consistent with prior studies in healthy human aging that have applied region of interest and voxel-based univariate analyses, showing relative preferential effects of aging on prefrontal and selective temporal brain areas (Raz et al., 1998; Good et al., 2001; Tisserand et al., 2002; Raz and Rodrique, 2006; Park and Reuter-Lorenz, 2009), but other regions have also shown volume reductions, including parietal cortex, cerebellum, and subcortical white matter. We did not observe age effects in regions comparable to the human parietal cortex and we did not investigate white matter differences in the current study, but we did observe small areas of reduction in the SSM VBM age-related pattern in the cerebellum. Future research should evaluate whether such cerebellar gray matter reductions are associated with behavioral effects of aging, including potential differences in motor learning or coordination.

It is also noteworthy that the pattern of age-related gray matter volume reductions in our study extended over several temporal brain regions, including the perirhinal and temporal association cortices. The MRI gray matter volume reductions may reflect age-related synaptic loss with reductions in dendritic arborization (Morrison and Hof, 2003; Burke and Barnes, 2006). There has been recent and growing interest in the effects of aging on the perirhinal cortex, which has important roles in higher-order cognitive processing of sensory information, contextual and associate learning, and the ability to discriminate between novel and familiar objects in the environment (Higuchi and Miyashita, 1996; McTighe et al., 2010; Ryan et al., 2012; Burke et al., 2014). Studies in both humans and rodent models of aging have shown differences with aging in the functional engagement of the perirhinal cortex with object recognition memory (Burke et al., 2010, 2014; Ryan et al., 2012). Our findings showing greater relative reductions in MRI gray matter volume, as part of the age-related network pattern, in the aged compared to young adult rats may reflect reduced connections with diminished dendritic arborization in the vicinity of the perirhinal cortex. Such relative reductions in volume in this brain region are consistent with reports of decreased function of this brain area in both functional MRI studies in humans and electrophysiological studies in the rat (Ryan et al., 2012; Burke et al., 2014). Further studies, using MRI scans to measure functional and structural connectivity in rodent models (e.g., Hsu et al., 2016) of aging are needed. Such studies may help identify how age-related alterations in the perirhinal cortex relate to differences in other regionally distributed brain structures, their connectivity, and the associated decrements with cognitive aging.

With the SSM network covariance method, we also observed areas with relatively increased gray matter in the vicinities of the midbrain, thalamus, septal nuclei, selective hippocampal, and somatosensory regions. Such relative increases may reflect relative preservation of volume as part of the regional covariance network pattern, but also may indicate areas where brain development may extend beyond the young adult age range in our study. It has been suggested, based on early histological work as well as more recent imaging studies, that some brain structures, including the hippocampal formation and others, in the rat, continue to enlarge through much of the adult lifespan (Diamond et al., 1975; Sullivan et al., 2006; Gaser et al., 2012; Sumiyoshi et al., 2014). Areas of relative increased gray matter volume in our aged sample may reflect such continued growth when compared to the group of young adult rats with an average age of 10.8 months. Although we used bootstrap resampling in our SSM network analysis to identify robust regions contributing to the observed age-related gray matter pattern, future work is needed with larger samples of young and old rats to investigate and further validate the major regions associated with aging in this rodent model. Our study included only male rats. Future work is also needed with both cross-sectional and longitudinal studies of structural MRI with SSM VBM that include both male and female rats to evaluate the naturally occurring regional network differences of brain volume throughout development and aging over the full adult lifespan. In this context, it will also be valuable to combine multiple neuroimaging modalities, including different types of structural, functional, and metabolic imaging in a multimodal framework to better understand how the brain is altered by age. Such research could help clarify how cognitive and brain aging is influenced by differences in functional and structural compensation over the lifespan. We used the Fischer 344 rat for our study of age-related differences in regional MRI network gray matter, as there have been decades of experience in investigating aging behavior in this rodent model. This work could also be extended to different rodent and other animal models, including those that develop spontaneous health conditions, to evaluate how the development of such comorbid conditions often associated with human aging, like hypertension (Kern et al., 2017; Willeman et al., 2019; Van Etten et al., 2020), impact the aging brain. In this case, further methodological development efforts will be important to help facilitate the harmonization of MRI and other imaging modalities with differing spatial resolutions to support such cross-species comparisons.

We also found that greater expression of the SSM MRI VBM age-related gray matter pattern was associated with poorer learning on the spatial Morris swim task in the young and aged groups combined. It is well established that spatial learning declines with age in rats (Burke and Barnes, 2006; Gallagher et al., 2015) and has linkages to aspects of age-related memory decline in humans (Alexander et al., 2012a). It is widely held that cognitive decline during aging is mediated by alterations in vulnerable brain structures, which can impact the neural systems and associated connectivity that support cognition (Li et al., 2001; Burke and Barnes, 2006; Park and Reuter-Lorenz, 2009). The brain is thought to have a reserve capacity that may, in part, account for individual differences in cognitive aging, but when sufficiently depleted, leads to the expression of cognitive impairment (Stern et al., 1995, 2005; Alexander et al., 1997). Unlike neurodegenerative diseases, like Alzheimer’s disease, the relatively subtle effects of aging on synaptic transmission and plasticity and cellular function and morphology may have a key role in the process leading to brain aging and the associated cognitive decline (Barnes, 2001; Morrison and Hof, 2003; Burke and Barnes, 2006).

Together, these findings suggest that the application of cross-cutting neuroimaging techniques, like SSM MRI VBM, can advance cognitive aging research with the potential for translating across species from human to non-human primate, to small animal models, and with back translation to humans. Clinical translational research has typically focused on the translation of findings with potential clinical relevance identified in small animal models that move toward expanded applications in large human intervention trials. By first using methods that are applied in human aging research to initially characterize comparable effects in small animal models, the ability to identify important and relevant regional targets for higher resolution molecular studies in rodents, which are anchored *a priori* in comparable brain regions showing human aging effects, may be enhanced. With this added ability for the *regional back translation* provided by such cross-cutting neuroimaging methods, there may be great potential for identifying regional targets in rodent models that offer greater promise in helping to elucidate the underlying mechanisms, for example through gene expression or epigenetic processes, leading to brain and cognitive effects with specific relevance to the human aging process. Using multivariate covariance methods, like SSM with VBM, has the potential to enhance our understanding of underlying mechanisms of brain aging and how new behavioral or pharmacological interventions designed to improve cognitive function during aging may benefit brain structure, function, and connectivity.

Our findings support the use of multivariate SSM VBM network analysis with high-resolution MRI for translational studies evaluating the effects of cognitive and brain aging in rodent models. That the pathological features of neurodegenerative diseases or other common medical conditions of human aging, such as Alzheimer’s disease and hypertension, are not naturally observed in the rat provides an important opportunity to evaluate aging effects on the brain without potential impacts of underlying developing disease common to human aging. Applying a multi-modal approach that uses multiple MRI scans to measure complementary aspects of brain structure, function, and connectivity in the same individual, in studies of both humans and non-human animal models, combined with multivariate network analyses, like SSM, may ultimately provide the greatest opportunity for defining a comprehensive neuroimaging profile of brain changes that occur during aging. This should advance our understanding of neural systems impacted by the aging process, while also helping to directly evaluate interventions across species that are designed to diminish or delay aging effects on specific neural targets, leading to effective amelioration of the cognitive decline associated with human brain aging.

## Conclusions

Using high-resolution structural MRI of the brain and a multivariate network model of covariance with SSM VBM, we investigated the regional pattern of gray matter volume in a rodent model of aging. The results suggest that aging in the rat is associated with a regionally distributed pattern of gray matter volume reductions, including in frontal and selective temporal brain regions, reflecting areas where aging effects have been previously observed in MRI studies of humans and non-human primates. Together, our findings suggest the application of cross-cutting neuroimaging techniques, like SSM MRI VBM network covariance analyses, can advance translational aging neuroscience research, extending from human to small animal models, with the potential for evaluating mechanisms and interventions for cognitive aging.

## Data Availability Statement

The raw data supporting the conclusions of this article will be made available by the authors, without undue reservation.

## Ethics Statement

The animal study was reviewed and approved by the University of Arizona Institutional Animal Care and Use Committee.

## Author Contributions

GA, CB, and TT contributed to study oversight, data collection, data preparation, analyses, interpretation, and manuscript preparation. LL, EY, LH, and MC contributed to data collection, data preparation, analyses, interpretation, and manuscript preparation. PB, KB, KC, and JM contributed to data preparation, analyses, interpretation, and manuscript preparation.

## Conflict of Interest

The authors declare that the research was conducted in the absence of any commercial or financial relationships that could be construed as a potential conflict of interest.
